# Considerations for the Design of a Physical Fitness Battery to Assess Adults with Intellectual Disabilities: Preliminary Reference Values for the SAMU DIS-FIT Study

**DOI:** 10.3390/ijerph17249280

**Published:** 2020-12-11

**Authors:** Ruth Cabeza-Ruiz

**Affiliations:** Department of Human Movement and Sport Performance, University of Seville, 41013 Seville, Spain; ruthcr@us.es

**Keywords:** disability, tool, health, life span, physical fitness, reference values

## Abstract

For the assessment of the health-related physical fitness (PF) of individuals with intellectual disabilities (ID), tools designed for people without disabilities have generally been used. Also, the results of these assessments have routinely been compared with the scores obtained by people without ID. The objectives of the present study are to present the rationale for the design of an assessment battery for PF, the so-called SAMU DIS-FIT battery, and to present the results obtained by the participants classified according to age, sex, and level of PF (physical fitness). The selection criteria for the tests that would make up the battery were: (i) utility, (ii) psychometric properties, (iii) easiness and diversity, (iv) simplicity of execution, (v) familiarity and motivation, and (vi) economy of resources. A cross-sectional study was designed to assess the PF of 261 individuals with ID. To interpret the results obtained by the participants, each of the quantitative variables of PF was categorized into three levels: lower-fit, mid-fit, and higher-fit. The findings of this study serve as a first step in establishing PF baseline values for individuals with ID.

## 1. Introduction

Individuals with intellectual disabilities (ID) generally show lower levels of physical fitness (PF) than those without disabilities of the same sex and age [1,2,3,4]. However, making comparisons between values obtained by individuals without disabilities and individuals with disabilities could imply a comparative disadvantage for the latter because the personal and social development as well as the characteristics of adults with and without ID are not similar. In general, studies describing the PF of individuals with ID are scarce or use small samples. Only studies carried out with people who participate in the Special Olympics World Games have many participants [5,6,7,8], but they do not provide the psychometric properties of the tests used, so the suitability of the tools used for the assessment of PF in individuals with ID is unknown. Hilgenkamp et al. have conducted studies with large samples of older people with severe or profound ID [9]. The results of their research show good to excellent values for the psychometric properties of the tests used. However, it was necessary to have a battery of tests with good reliability and feasibility for younger adults with mild or moderate ID as they constitute the largest group of individuals with ID.

In many cases, tests designed for people without ID, which have passed validity tests, have been used to assess PF in individuals with ID [10,11,12,13,14]. In some cases, these tests have been evaluated for their reliability in the ID population but only with small samples [15] or older people [16]. The reliability of some isolated tests has also been evaluated [17], and, to a lesser extent, original tests have been adapted to be performed by individuals with ID, providing feasibility values [9,18,19].

In September 2017, the SAMU Foundation (Seville, Spain), an organization that assists people with disabilities, and the Department of Human Movement and Sport Performance of the University of Seville (Seville, Spain) were interested in carrying out a joint study (University of Seville 3054/0780) on constructing PF tests for adults with ID. The study came up with a battery of tests to assess PF in individuals with ID, adapted to their characteristics and with appropriate psychometric criteria. To this end, it was necessary to review the scientific literature to find previous studies and select PF assessment tests that could form a feasible, reliable, and valid battery for individuals with ID. The results of these meetings and literature studies led the research team to formulate the following long-term objectives: (i) to design a PF assessment tool for persons with ID that would provide reliable data on their fitness status; (ii) to design a PF assessment tool for individuals with ID that would take into account the characteristics of this population; and (iii) to provide a description of the PF status of individuals with ID according to age, sex, and fitness level that would serve as a guide for other researchers and clinicians and that would be based on the results of a large sample of persons with ID.

Two years later, the battery, called the SAMU DIS-FIT, was designed and tested. Although the results on the suitability of the battery have previously been published [20,21], the criteria that were taken into account by the research team to select the different tests based on the characteristics of the population with ID have not yet been shared with the scientific community. Neither were the results obtained by the participants of the SAMU DIS-FIT study available to date. Therefore, the objectives of the present study are: (i) to describe the qualitative aspects that led the research team to select the tests that would make up the battery for the assessment of PF in individuals with ID and (ii) to show the results obtained by the participants according to sex, age, and level of physical condition.

## 2. Materials and Methods

### 2.1. Rationale for the Test Battery Used

The research team, consisting of four sports science and physical activity professionals, two psychologists, and one social worker with more than 5 years of experience working with individuals with ID, met to design a battery for the assessment of PF in adults with ID. To do so, it was necessary to hold a brainstorming session to gather all the aspects that the team believed were important when working with individuals with ID. In addition, a review of the literature was conducted to identify studies related to the objective of the project that could be used to select and analyze those tests that meet the psychometric criteria. Once the ideas were compiled, they were grouped into categories related to motor aspects; psychobehavioral aspects; group-specific needs; consistency of the tool; and aspects related to material, human, and economic resources.

The project was divided over two years by the SAMU Foundation and the University of Seville. During the first 3 months, the literature review was carried out, and the inclusion criteria for the tests that would form part of the battery were established. Subsequently, working meetings of the multidisciplinary team were held to design the final tool. Simultaneously, a group of last-year Sport Science students was selected to participate in the study as research fellows. The chosen students were given a training course to learn how to handle and apply the PF assessment battery and were called to participate as evaluators in the pilot study.

To achieve the research objectives, a large sample of individuals with ID was needed. After pre-selecting and recruiting the sample, the next step was to design the final PF assessment instrument that was appropriate for the research requirements and respectful of the characteristics of individuals with ID. Assessing individuals with disabilities is often challenging because of the particularities of the disabilities. The different ways of receiving, processing, and interpreting information or aspects related to motivation, attention, and communication are a major challenge for data collection and the certainty that the data are consistent with reality when working with persons with disabilities [22,23].

As a result of these meetings, the following criteria were established for the inclusion of a test in the fitness test battery:

(1) Utility criteria

The first element that was considered when designing the battery was that it should comply with the recommendations of the American College of Sports Medicine [24]. To this end, the assessment of the PF components directly related to health, and not merely to fitness-related skills, was studied, and a combination of six tests was proposed to evaluate the four fundamental components of PF [16]: body composition, flexibility, muscle strength, and cardiorespiratory fitness. Although body balance is regarded as a fitness-related skill and not a health-related fitness component [25], without the ability to maintain body balance it is impossible to perform any other motor action, so a test to assess dynamic balance was included in the battery.

(2) Psychometric criteria: validity, reliability, and feasibility

Once the most relevant fitness components had been selected, psychometric criteria were taken into account for the selection of the tests. In order to design a solid, useful test battery based on objective criteria of repeatability, the instrument had to be built on the basis of validity, reliability, and feasibility to be included in the battery [23]. In this way, a tool would be obtained that provides accurate information about the PF levels of individuals with mild or moderate ID. Validity is the adequacy and appropriateness of a test to accurately measure what it is trying to measure (to learn more read Yun & Ulrich, 2002) [26]. In the battery, one test was included if there were previous studies on the validity of the instrument. The reliability of a tool refers to its ability to obtain the same results when measuring the same phenomenon again. To know whether an instrument measures the same thing at different times, the intraclass correlation coefficient could be used, among other measures. Finally, feasibility is the rate of successful cases—that is, the percentage of individuals who perform the test properly, which entails not only understanding the test but also executing it correctly [27].

The following steps were taken to meet these objectives:

(a). The selected tests were those previously supported by validity studies.
(b). If the previous criterion was met, reliability and feasibility criteria were also applied, either by reviewing previous studies undertaken with individuals with ID or by carrying out pilot studies by the research team.

(3) Criteria of simplicity and diversity of instructions

Individuals with ID are characterized by a different memory and attention capacity and by a different way of processing, retrieving, or integrating information. The way in which the instructions are presented conditions the way in which the subjects will understand the dynamics of a test and, subsequently, the way they perform it [18,23]. In tests for which it is necessary to provide extensive and detailed information because of the technical complexity of the movement, individuals with ID will encounter barriers to understanding and memorization. That is why it is necessary to offer simplified information—that is, to give a participant only the necessary information to perform the test properly. For example, to correctly execute a maximum vertical jump, it is sufficient to ask a participant to jump as high as possible, without including information on specific technical aspects, such as the degree of knee flexion or arm coordination. In addition to verbal information, it is essential that evaluators give demonstrations of every test to each of the participants—sometimes more than once. It is possible that, in some cases, it is necessary to use pictograms and other forms of communication, such as sign language.

(4) Criterion of easiness of motor response

Even if the information received is simple and the individuals with ID can understand the procedure of the tests following the techniques required, it is also necessary that these tests do not involve a complex motor act—that is, that the test is also simple in relation to its execution [23]. It is well known that people with disabilities lead sedentary lives [28,29,30,31] as they encounter numerous barriers to participating in regular physical activities [8], so their level of motor skills is often limited. Although some tests may seem simple in their description, sometimes the execution of these tests involves taking into account numerous elements that could invalidate the results. An example of this is the Sit and Reach Test, in which the assessed persons must maintain trunk flexion while extending both arms without bending their knees.

(5) Familiarity and motivation criteria

This aspect refers to the requirement that the places, the instruments, and the assessment staff are, as far as possible, familiar to the participants [23]. Taking into account the psychological and behavioral characteristics of individuals with ID, the research team recommended that the assessments should ideally be carried out in the participants’ care centers, since it could improve the outcome of the assessments when the participants feel more comfortable and confident [32,33]. The researchers also advised that the assessments be done in a group, since this way the participants feel accompanied and motivated by their peers, which generates familiarity and confidence, creating a more favorable work environment, in which the assessments do not seem like an examination but rather a game.

(6) Cost criterion

Some tests for assessing PF are considered to be more objective than others. In general, these are tests that are carried out under strictly controlled conditions, with highly sophisticated instruments, and in laboratories dedicated to the assessment of the physical and physiological capacities of humans. However, these types of instruments represent a high-cost investment that is not available to most organizations that attend to individuals with disabilities. Moreover, the use of these instruments requires specific training of the assessment staff, and these professionals must often be health care personnel [22]. For these reasons, PF assessment tests with less complex instruments are more affordable to professionals who work with individuals with disabilities outside of universities or research centers. These instruments are less complex and expensive than laboratory equipment and therefore can be operated by anyone with minimal training.

In addition to material and human resources, time management was another important aspect that the researchers considered when designing the battery. As a main objective, it was proposed that the complete assessment of a person should not exceed 45 min. However, in the present study, strategies are proposed for assessing groups of five people in approximately 1 h. These time management principles aim not only at facilitating the measurements for the evaluators but also at avoiding the physical and psychological fatigue of the participants.

### 2.2. Design

A cross-sectional design was employed. This study is part of a larger project with a test-retest design in which the reliability and feasibility of the final battery were tested.

### 2.3. Participants

To recruit as many participants as possible, the heads of the SAMU Foundation contacted the directors of 15 local associations in Seville (Spain) that attend to individuals with ID to invite them to participate. In these contacts, the procedures and objectives of the study were explained to the directors of the interested centers. Subsequently, each center director contacted their clients and their families to invite them to participate. An information sheet was used. Those individuals with ID who showed interest in participating were given a consent form to be signed by each participant and co-signed by their legal guardians if necessary. Of the 15 organizations consulted, 12 agreed to collaborate. In addition to the participation consent, all participants were required to submit a medical authorization, signed by their referring physician, that confirmed their ability to carry out physical activity without risk to their health. Of the 753 individuals with ID contacted, 300 were selected. Finally, the study included 261 people between 18 and 65 years old with mild or moderate ID (82 women and 179 men), of whom 37 had Down syndrome (11 women and 26 men).

The biomedical committee of the competent government was asked to evaluate the research project (Biomedical Research Committee of the Junta de Andalucía, Andalucía, Spain), which was approved with internal code 0316-N-15. The study was conducted in accordance with the Declaration of Helsinki.

### 2.4. Materials

#### The SAMU DIS-FIT battery

After the application of the criteria described above all those tests that did not meet these criteria were discarded. Finally, the battery was composed of the following tests: body composition (body mass index and waist circumference), muscular strength (upper, middle, and low body strength), flexibility, motor fitness (dynamic balance), and cardiorespiratory fitness. The procedures for performing the tests can be read elsewhere [20,21].

(1) Body composition

The body mass index (BMI) was selected for the assessment of body composition for its reliability and feasibility in the ID population [19]. The BMI has shown a good correlation with other instruments for measuring body composition [34], is simple to carry out, does not require complex equipment, and is cost-effective.

Besides the use of a general body fat index, the waist circumference (WC) was measured as an index of the visceral adipose tissue, located in the middle of the body. The WC has shown strong correlations with other body composition measures that provide good validity results [34]. The results of feasibility and reliability tests have shown good values in individuals with severe intellectual and sensory disabilities [19], and its use is simple and affordable, so the research team decided to include it in the battery. Body composition tests do not require the active participation of the participants, so they do not involve demands on cognitive or motor functions.

(2) Muscular strength

To assess muscle strength, three different tests were selected: (i) Grip Strength (GS), (ii) 30 s Sit Up (SUP), and (iii) the Timed Stand Test (TST). The use of three tests was justified by the need to assess different manifestations of strength and different muscle groups as a basis for determining a person’s PF [2,15].

The GS test measures the muscle tension that can be generated by the muscles of the hand and forearm and is related to the ability to perform daily tasks and to the nutritional status of a person. This test has previously been tested for its psychometric properties of reliability and feasibility in older individuals with severe ID, obtaining good results [16]. The validity of the test has shown positive results in older adults [10].

The SUP estimates the endurance strength of the abdominal muscles and the hip flexors. For this purpose, the test proposed by Skowroński et al. was adapted [35] so that it records a repetition if the subjects manage to touch their knees with their hands. This test has obtained good indices of validity [36] and reliability [32] in individuals with ID, but previous feasibility studies are lacking, so it was decided to include it in the battery in order to find out its suitability in individuals with ID.

For the assessment of lower limb strength, the 10-repetition TST was used because it was thought that individuals with ID would have better results in brief, targeted tests in which they can manage their effort (10 repetitions as quickly as possible) more easily than in longer tests. The validity and reliability of this test have previously been tested in people with different diseases [37]. No previous feasibility studies were found in adults with moderate ID. However, Hilgenkamp, Van Wijck, and Evenhuis [16] found moderate values of feasibility and good reliability in the 30 s TST in elderly adults with severe ID.

All three tests require cost-effective instruments, as well as low cognitive and motor involvement of the participants in performing the tests, so the research team selected them to be included in the final battery.

(3) Dynamic balance

No static tests were used to assess body balance. In previous studies conducted with populations with similar ID characteristics, the reliability results of the One-Leg Stand test were low [38], so tests on one foot were discarded and replaced by more natural dynamic tests. The selected test was a modification of the Timed Up and Go (TUAG) test [39]. The reliability of the test has previously been studied in individuals with ID [40], but in the test, the subjects moved at “a comfortable speed.” In our opinion, it is easier and more reliable to move “as fast as possible” than to prescribe a comfortable speed. Validity tests in previous studies have obtained excellent results for people with chronic stroke [41]. No previous feasibility data were found, but the test was included with the aim of assessing its suitability in the population with ID, as well as for its simplicity and reduced cost.

(4) Flexibility

Flexibility would be assessed with the Deep Trunk Flexibility (DTF) test, which measures the range of motion in the spine and hip during deep trunk flexion. Although no previous studies of validity, feasibility, or reliability of the test were found, its choice was based on the fact that its execution is similar to the execution of everyday tasks, such as picking up objects from the ground, making it a simple and cost-effective test.

(5) Cardiorespiratory fitness

Cardiorespiratory fitness is one of the main components of PF. The 6-Minute Walk Test (6MWT) has previously been tested in different studies to determine its reliability and validity in populations with ID, showing excellent values both in adults [17,32] and in adolescents [42]. However, these studies have been carried out with small samples and without differentiating participants according to sex. Regarding the feasibility of the test, Wouters et al. [18] found positive results in children with ID. The equipment needed to perform the test is inexpensive, and the test requires participants to perform a regular activity of daily life, so the test was included in the final battery.

### 2.5. Duration and Order of Test Administration

The research team went in groups of five people with the testing material to each of the care centers to assess the residents who had given their consent to participate. The assessments were always carried out between 10 a.m. and 2 p.m. The tests were conducted between January 2018 and December 2018. This was one of the great challenges of the research group, since all the staff had to organize themselves in order not to miss the weekly evaluations. In the first half of the year, 160 people were assessed, and in the second half, after the summer months, 101 people. On average, about 10 subjects were assessed per week.

The duration of the tests depended on the characteristics of each participant. It was recommended that there were several evaluators in order to make a wheel of measurements that allowed the evaluation of an average of five people in 1 h. Owing to the attentional and memory characteristics of people with ID, it was not possible to give the information about how to perform the tests collectively, so it was necessary to explain the protocol to each participant individually. Therefore, the total battery duration was 30–45 min per person. Explanations, demonstrations, and attempts are included in that period of time.

Regarding the order of the application of the tests, the recommendation is to perform the body composition test at the beginning and the cardiorespiratory fitness test at the end. The remaining tests can be applied in any order or counterbalancing, as none of them involve such a significant effort that they can influence the performance of the subsequent tests. As a guideline, this could be the order of a complete assessment:

(1) Body composition (5–8 min)

(2) Timed Up and Go (2–3 min)

(3) Deep Trunk Flexibility (2–3 min)

(4) Grip Strength (3 min)

(5) Sit Up (5 min)

(6) Timed Stand Test (5–10 min)

(7) 6-Minute Walk Test (10 min)

### 2.6. Data Analysis

To categorize each of the quantitative variables of PF into three levels, we used 95% of the mean confidence interval (mean 95%CI) for each group (men, women) and for each age range (young [< 30 years old], middle-aged [30–50 years old], and old [>50 years old]). Based on the mean 95%CI, the values above and below this interval were established, following Bohannon’s indications [43]. In this way, the results were grouped into three categories: lower-fit (results under mean 95%CI), mid-fit (mean 95%CI), and higher-fit (over mean 95%CI). The values obtained were rounded to the nearest whole number. Finally, to calculate the percentages and number of subjects in each PF category, custom tables were designed for each variable, sex, and age range. All these analyses were performed in SPSS, PASW Statistics 18 (IBM, Inc., Armonk, NY, USA).

## 3. Results

**Figure 1:**
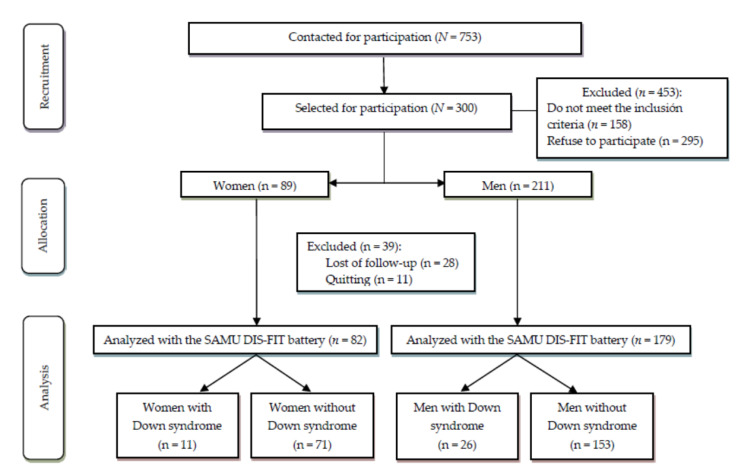
Study flowchart.

Figure 1 shows the flow chart with the whole sample participating in the project. Concerning the achievement of the first objective of this study, all the criteria that were established for the design of the battery have been explained. The results of reliability and feasibility have been published previously [20,21]. The results on PF obtained by the participants according to sex, age, and level of PF can be found in Table 1, Table 2, Table 3, Table 4, Table 5 and Table 6.

The custom tables resulted in three categories for the male participants: younger (n = 46), middle-aged (n = 111), and older (n = 22). For women, the sample sizes for each category were as follows: younger (n = 23), middle-aged (n = 47), and older (n = 12).

In the male group, more than half of the young and middle-aged (18–50 years) participants had a BMI > 27 kg/m^2^. More than 72% of those over 50 years old had a BMI > 25 kg/m^2^. All the male participants had WC results > 89 cm. In the group of young women, more than 50% presented a BMI above 25 kg/m^2^ and a WC > 83 cm. Almost 70% of the women between 30 and 50 years presented a BMI > 30 kg/m^2^, and more than 50% a WC > 96 cm. Approximately 85% of the old women had a BMI above 24 kg/m^2^ and a WC of more than 83 cm.

As for the variables related to muscle strength, dynamic balance, and flexibility, approximately 50% of the young and middle-aged men were in the higher-fit range. However, only 17 out of 46 young men were in that category for the cardiorespiratory fitness test. Between 36.4% and 40.9% of men over the age of 50 were in the higher-fit category for the GS, SUP, TST, and DTF tests. Only 3 (13.6%) and 2 (9.1%) people had the highest values in the TUAG and 6MWT tests, respectively.

Between 30.4% and 43.5% of the younger women obtained higher-fit values in the GS, SUP, TST, and DTF tests. However, these percentages were lower in the TUAG and 6MWT tests (17.4% and 26.1%, respectively). The tests in which more middle-aged women fell in the higher-fit category were the SUP and TST (36.2%) and 6MWT (38.2%) tests. In the older women group, all the PF variables were in the mid-fit and lower-fit categories.

## 4. Discussion

This is the first study to present PF values of adults with ID categorized into lower-fit, mid-fit, and higher-fit groups. It is necessary to point out that in this study, no attempt was made to relate belonging to one of these categories to a better health status. The author has just established three levels of PF according to the results of the participants themselves, thus avoiding a comparison with individuals without disabilities. The results of PF tests in any type of population need an interpretation of the data to give meaning to the values achieved by a particular person. In this type of studies, the results obtained by individuals with disabilities are usually compared with those obtained by individuals without disabilities [8,44]. This can be interesting if the reference values that establish relationships between the levels of fitness and health are included. However, because the personal and social development as well as the characteristics of individuals with and without ID are not similar, it seems inappropriate to make comparisons between both groups. If this is done, a person with ID who has good values within his or her group will present a low fitness level when compared with individuals with typical development, just as an older person will have lower PF results than a younger person of the same sex.

Since there are no previous studies conducted on individuals with ID that present their results categorized by PF level, it is necessary to discuss our findings by making comparisons with the results of other studies in which central tendency data, such as the mean, are presented and in which the same assessment instruments have been used as in the present work.

In a study conducted by Boer and Moss [32], in which PF was assessed in 43 adults with Down syndrome aged 18–45 years, average BMI values of 30.3 kg/m^2^ were found. In the same work, the values obtained by the participants in the 6MWT ranged from 513 to 578 m. Both results are similar to those of the present study. However, the participants of the SAMU DIS-FIT study present average values of 30 kg in the GS tests. This difference may be due to the fact that men and women were included in the same group in Boer and Moss’s study, so their results are lower. Hilgenkamp, van Wijck, and Evenhuis [4] obtained values close to 30 kg in GS tests performed on a large sample of older men with severe ID and values between 20.08 and 21.34 kg in a group of women with the same characteristics.

Guerra et al. [17] performed cardiorespiratory fitness tests on 46 individuals with mild, moderate, and severe ID. The results from participants with mild and moderate ID showed means between 449.6 and 531.7 m in the 6MWT. Although their results are not presented for men and women separately, they are similar to those obtained in the present work.

In the 10-repetition TST, Cuesta-Vargas, Paz-Lourido, and Rodriguez [8] assessed 266 individuals with ID with a mean age of 31.1 years (187 males and 79 females) recruited from the Spanish Special Olympics Games. The men obtained mean values of 19.9 ± 9.5 s, while the women completed the test with mean values of 22.8 ± 9.7 s. Their results are similar to those obtained in the present study, both in the men’s and women’s groups.

Although no similar studies that used DTF, TUAG, and SUP tests have been found the present results cannot be compared with those in the literature, it is necessary to emphasize that the findings achieved by other researchers in assessment tests of GS, 6MWT, TST, and BMI are similar to those of the SAMU DIS-FIT study, which could indicate that the reference values presented here are not different from those obtained by individuals with ID who participated in other studies.

In light of the results of this study, it should be noted that a high percentage of people with mild or moderate ID of both sexes present medium and low PF values. This low PF impairs the quality of life and lowers the life expectancy of people with ID more than being obese [45] and leads to high costs of families and health systems. However, other authors state that despite having low fitness, individuals with ID who minimally improve their PF gain significant health benefits [44]. In this sense, this paper may be useful in guiding clinicians to design and run physical exercise programs to improve the PF level of individuals with ID, so that minimally improving the score of a given PF component could significantly improve a person’s level of health. For example, an old man who scores 20 kg in the GS test would be in the lower-fit level, but if he managed to improve his performance by 3 kg and be in the mid-fit level, he could be significantly improving his health. To this end, some authors have suggested different types of physical exercise programs that can generate benefits in PF of individuals with intellectual disabilities [46].

One of the most relevant aspects of knowing the PF of adults with ID is to make predictions regarding their functional performance based on their own results. In this respect, Terblanche and Boer [36] studied the functional fitness capacity of 371 adults with Down syndrome. The researchers found that the capacity most closely related to functional independence for activities of daily living was leg strength, both for men and women, but also grip strength. In the same vein, other studies analyzed the relationship between different PF components and gait parameters in 31 adults with ID. They found that body composition was related to gait performance at comfortable speed but that muscular endurance and body balance were related to gait performance at fast speed [47]. This is important because a decrease in walking speed has been associated with a lack of autonomy, disability, risk of falls, and mortality. In a longitudinal study conducted with 601 older adults with ID [48], different PF components (manual dexterity, balance, gait speed, muscular endurance, and cardiorespiratory fitness) were related to PF decline after 3 years, so PF turns out to be a fundamental aspect in maintaining activities of daily living and autonomy in adults with ID. In this sense, using the SAMU DIS-FIT, and making comparisons with the values presented here, can help individuals with ID, their families and clinicians to know their fitness level and make predictions based on those results in order to carry out physical activity programmes that improve their level of autonomy in the future.

The present study’s limitations are related mainly to the size of the sample. On the one hand, although a total sample of 261 individuals with ID is substantial, it is not large enough to establish reference values for the whole population with ID. It is therefore necessary to stepwise increase the number of individuals with ID who are assessed with the same instruments so that the database can be expanded and bigger sample sizes can be reached in order to establish PF reference values for the ID population. On the other hand, because of the categorization of the results into age groups, the sample sizes of the groups, especially those with older participants, became too small to be representative of people with ID.

Finally, the scientific field must establish PF reference values for the population with ID in order to know their capacities without falling into the error of demanding the same levels of PF as those established for adults without disabilities. Knowing the limitations and potential of individuals with ID will make it possible to set up respectful health improvement programs that are suited to their particularities and characteristics.

## Figures and Tables

**Table 1:** Younger men (<30 years old) results for each variable categorized in 3 physical fitness levels.

N = 179; n = 46 (N% = 25.7)		Higher-Fit	Mid-Fit	Lower-Fit
**BMI (kg/m^2^)**	Cutoff point Mean Nº of people (n%)	<27 23.1 18 (39.1%)	27–30 28.6 11 (23.9%)	>30 34 17 (37%)
**WC (cm)**	Cutoff point Mean Nº of people (n%)	<91 81.9 20 (43.5%)	91–99 96.4 8 (17.4%)	>99 108.9 18 (39.1%)
**GS (kg)**	Cutoff point Mean Nº of people (n%)	>34 41.7 19 (41.3%)	28–34 30.1 11 (23.9%)	<28 21.8 16 (34.8%)
**SUP (rep)**	Cutoff point Mean Nº of people (n%)	>20 23.9 19 (41.3%)	16–20 17.4 11 (23.9%)	<16 11.1 16 (34.8%)
**TST (s)**	Cutoff point Mean Nº of people (n%)	<16 12.57 22 (47.8%)	16–20 17.45 8 (17.4%)	>20 25.29 16 (34.8%)
**TUAG (s)**	Cutoff point Mean Nº of people (n%)	<4 3.4 23 (50%)	4–5 4.6 13 (28.3%)	>5 5.9 10 (21.7%)
**DTF (cm)**	Cutoff point Mean Nº of people (n%)	>40 45.1 22 (47.8%)	35–40 36.8 8 (17.4%)	<35 27.8 16 (34.8%)
**6MWT (m)**	Cutoff point Mean Nº of people (n%)	>595 673.4 17 (37.0%)	532–595 571.5 9 (19.6%)	<532 466.4 20 (43.5%)

BMI, body mass index; WC, waist circumference; GS, grip strength; SUP, 30 s Sit Up; TST, timed stand test; TUAG, timed up and go; DTF, Deep trunk flexibility; 6MWT, 6 min walk test.

**Table 2:** Middle-aged men (30–50 years old) results for each variable categorized in 3 physical fitness levels.

N = 179; n = 111 (N% = 62)		Higher-Fit PF	Mid-Fit	Lower-Fit
**BMI (kg/m^2^)**	Cutoff point MeanNº of people (n%)	<27 23.6 46 (41.4%)	27–30 28.6 28 (25.2%)	>30 34.5 37 (33.3%)
**WC (cm)**	Cutoff point Mean Nº of people (n%)	<96 86.7 50 (45%)	96–101 98.7 13 (11.7%)	>101 110.6 48 (43.2%)
**GS (kg)**	Cutoff point MeanNº of people (n%)	>32 38.5 51 (45.9%)	29–32 30.0 8 (7.2%)	<29 23.3 52 (46.8%)
**SUP (rep)**	Cutoff point Mean Nº of people (n%)	>19 22.2 56 (50.4%)	16–19 17.2 14 (12.6%)	<16 11.3 41 (37%)
**TST (s)**	Cutoff point Mean Nº of people (n%)	>20 24.9 43 (38.7%)	18–20 18.9 14 (12.6%)	<18 14.8 54 (48.6%)
**TUAG (s)**	Cutoff point Mean Nº of people (n%)	<4 3.5 47 (42.4%)	4–5 4.5 40 (36%)	>5 6.2 24 (21.6%)
**DTF (cm)**	Cutoff point Mean Nº of people (n%)	>36 41.4 51 (45.9%)	33–36 34 18 (16.2%)	<33 26.8 42 (37.9%)
**6MWT (m)**	Cutoff point Mean Nº of people (n%)	>538 607.5 48 (43.2%)	503–538 519.3 18 (16.2%)	<503 430.8 45 (40.6%)

BMI, body mass index; WC, waist circumference; GS, grip strength; SUP, 30 s Sit Up; TST, timed stand test; TUAG, timed up and go; DTF, Deep trunk flexibility; 6MWT, 6 min walk test.

**Table 3:** Older men (>50 years old) results for each variable categorized in 3 physical fitness levels.

N = 179; n = 22 (N% = 12.3)		Higher-Fit	Mid-Fit	Lower-Fit
**BMI (kg/m^2^)**	Cutoff point Mean Nº of people (n%)	<25 20.9 6 (27.3%)	25–30 27 7 (31.8%)	>30 32.9 9 (40.9%)
**WC (cm)**	Cutoff point Mean Nº of people (n%)	<89 77.2 6 (27.3%)	89–101 95.6 7 (31.8%)	>101 107.4 9 (40.9%)
**GS (kg)**	Cutoff point Mean Nº of people (n%)	>28 31.2 8 (36.4%)	23–28 24.9 6 (27.3%)	<23 19.5 8 (36.4%)
**SUP (rep)**	Cutoff point MeanNº of people (n%)	>17 20.3 9 (40.9%)	13–17 15.3 5 (22.7%)	<13 10 8 (36.4%)
**TST (s)**	Cutoff point Mean Nº of people (n%)	<18 15.7 8 (36.4%)	18–24 19.9 7 (31.8%)	>24 29.1 7 (31.8%)
**TUAG (s)**	Cutoff point Mean Nº of people (n%)	<4 3.7 3 (13.6%)	4–5 4.6 11 (50%)	>5 5.9 8 (36.4%)
**DTF (cm)**	Cutoff point Mean Nº of people (n%)	>35 40.1 8 (36.4%)	26–35 29.8 8 (36.4%)	<26 17.6 6 (27.2%)
**6MWT (m)**	Cutoff point MeanNº of people (n%)	>575 620.3 2 (9.1%)	457–575 508.9 11 (50%)	<457 428.3 9 (40.9%)

BMI, body mass index; WC, waist circumference; GS, grip strength; SUP, 30 s Sit Up; TST, timed stand test; TUAG, timed up and go; DTF, Deep trunk flexibility; 6MWT, 6 min walk test.

**Table 4:** Younger women (<30 years old) results for each variable categorized in 3 PF levels.

N = 82; n = 23 (N% = 28)		Higher-Fit	Mid-Fit	Lower-Fit
**BMI (** **kg/m^2^)**	Cutoff point Mean Nº of people (n%)	<25 22.3 8 (34.8%)	25–31 27.7 9 (39.1%)	>31 37 6 (26.1%)
**WC (cm)**	Cutoff point Mean Nº of people (n%)	<83 72.8 8 (34.8%)	83–96 90.5 7 (30.4%)	>96 105.8 8 (34.8%)
**GS (kg)**	Cutoff point Mean Nº of people (n%)	>26 30.7 8 (34.8%)	20–26 23 5 (21.7%)	<20 17 10 (43.5%)
**SUP (rep)**	Cutoff point Mean Nº of people (n%)	>18 21.7 7 (30.4%)	14–18 15.7 11 (47.9%)	<14 11 5 (21.7%)
**TST (s)**	Cutoff point Mean Nº of people (n%)	<18 15.2 10 (43.5%)	18–23 20.36 (26.1%)	>23 27.8 7 (30.4%)
**TUAG (s)**	Cutoff point MeanNº of people (n%)	<4 3.5 4 (17.4%)	4–5 4.4 14 (60.9%)	>5 6.4 5 (21.7%)
**DTF (cm)**	Cutoff point Mean Nº of people (n%)	>38 43.2 7 (30.4%)	31–38 35.1 10 (43.4%)	<31 21.9 6 (26.1%)
**6MWT (m)**	Cutoff point Mean Nº of people (n%)	>551 624.7 6 (26.1%)	457–551 502.2 12 (52.2%)	<457 400.4 5 (21.7%)

BMI, body mass index; WC, waist circumference; GS, grip strength; SUP, 30 s Sit Up; TST, timed stand test; TUAG, timed up and go; DTF, Deep trunk flexibility; 6MWT, 6 min walk test.

**Table 5:** Middle-aged women (30–50 years old) results for each variable categorized in 3 PF levels.

N = 82; n = 47 (N% = 57.3)		Higher-Fit	Mid-Fit	Lower-Fit
**BMI (kg/m^2^)**	Cutoff point Mean Nº of people (n%)	<30 25.2 17 (36.2%)	30–34 32.2 13 (27.6%)	>34 39.5 17 (36.2%)
**WC (cm)**	Cutoff point Mean Nº of people (n%)	<96 85.7 20 (42.5%)	96–105 101.3 7 (15%)	>105 115.3 20 (42.5%)
**GS (kg)**	Cutoff point Mean Nº of people (n%)	>23 28.4 14 (29.8%)	19–23 20.6 16 (30%)	<19 15.5 18 (38.2%)
**SUP (rep)**	Cutoff point Mean Nº of people (n%)	>16 21.1 17 (36.2%)	13–16 14.5 14 (29.8%)	<13 8.4 16 (34%)
**TST (s)**	Range Mean Nº of people (n%)	<20 15.4 17 (36.2%)	20–23 21.6 15 (31.9%)	>23 28.1 15 (31.9%)
**TUAG (s)**	Cutoff point Mean Nº of people (n%)	<4 3.7 11 (23.4%)	4–5 4.5 22 (46.8%)	>5 6.6 14 (29.8%)
**DTF (cm)**	Cutoff point Mean Nº of people (n%)	>35 40.4 16 (34%)	31–35 32.5 15 (32%)	<31 26.8 16 (34%)
**6MWT (m)**	Cutoff point Mean Nº of people (n%)	>489 18 (38.2%)	444–489 12 (25.6%)	<444 17 (36.2%)

BMI, body mass index; WC, waist circumference; GS, grip strength; SUP, 30 s Sit Up; TST, timed stand test; TUAG, timed up and go; DTF, Deep trunk flexibility; 6MWT, 6 min walk test.

**Table 6:** Older women (>50 years old) results for each variable categorized in 3 PF levels.

N = 82; n = 12 (N% = 14.6)		Higher-Fit	Mid-Fit	Lower-Fit
**BMI (kg/m^2^)**	Cutoff point Mean Nº of people (n%)	<24 20.8 2 (16.7%)	24–31 26.3 7 (58.3%)	<31 35.6 3 (25%)
**WC (cm)**	Cutoff point Mean Nº of people (n%)	<83 77.9 2 (16.7%)	83–96 86.8 7 (58.3%)	>96 103.8 3 (25%)
**GS (kg)**	Cutoff point Mean Nº of people (n%)	>23 30.7 1 (8.3%)	17–23 20.5 8 (66.7%)	<17 15.1 3 (25%)
**SUP (rep)**	Cutoff point Mean Nº of people (n%)	>21 29 2 (16.7%)	10–21 16.8 7 (58.3%)	<10 7 3 (25%)
**TST (s)**	Cutoff point Mean Nº of people (n%)	<18 16.8 3 (25%)	18–23 20.1 7 (58.3%)	>23 29.2 2 (16.7%)
**TUAG (s)**	Cutoff point Mean Nº of people (n%)	<4 3.2 1 (8.3%)	4–6 4.7 7 (58.3%)	>6 7 4 (33.4%)
**DTF (cm)**	Cutoff point Mean Nº of people (n%)	>39 42.9 4 (33.4%)	29–39 36.2 5 (41.7%)	<29 22.8 3 (25%)
**6MWT (m)**	Cutoff point MeanNº of people (n%)	>519 546.6 3 (25%)	430–519 501.6 5 (41.7%)	<430 387.24 (33.3%)

BMI, body mass index; WC, waist circumference; GS, grip strength; SUP, 30 s Sit Up; TST, timed stand test; TUAG, timed up and go; DTF, Deep trunk flexibility; 6MWT, 6 min walk test.
